# Alzheimer’s disease and infectious agents: a comprehensive review of pathogenic mechanisms and microRNA roles

**DOI:** 10.3389/fnins.2024.1513095

**Published:** 2025-01-07

**Authors:** Seyyed Sam Mehdi Hosseininasab, Rasoul Ebrahimi, Shirin Yaghoobpoor, Kiarash Kazemi, Yaser Khakpour, Ramtin Hajibeygi, Ashraf Mohamadkhani, Mobina Fathi, Kimia Vakili, Arian Tavasol, Zohreh Tutunchian, Tara Fazel, Mohammad Fathi, Mohammadreza Hajiesmaeili

**Affiliations:** ^1^Critical Care Quality Improvement Research Center, Loghman Hakim Hospital, Shahid Beheshti University of Medical Sciences, Tehran, Iran; ^2^School of Medicine, Shahid Beheshti University of Medical Sciences, Tehran, Iran; ^3^School of Medicine, Tehran University of Medical Sciences, Tehran, Iran; ^4^Liver and Pancreatobiliary Diseases Research Center, Digestive Diseases Research Institute, Shariati Hospital, Tehran University of Medical Sciences, Tehran, Iran; ^5^Student Research Committee, School of International Campus, Guilan University of Medical Sciences, Tehran, Iran; ^6^Department of Anesthesiology, Critical Care Quality Improvement Research Center, Shahid Modarres Hospital, Shahid Beheshti University of Medical Sciences, Tehran, Iran

**Keywords:** Alzheimer’s disease, pathogen, viral infection, bacterial infection, parasite

## Abstract

Alzheimer’s Disease (AD) is the most prevalent type of dementia and is characterized by the presence of senile plaques and neurofibrillary tangles. There are various theories concerning the causes of AD, but the connection between viral and bacterial infections and their potential role in the pathogenesis of AD has become a fascinating area of research for the field. Various viruses such as *Herpes simplex virus 1* (HSV-1), *Epstein–Barr virus* (EBV), Cytomegalovirus (CMV), influenza viruses, and Severe Acute Respiratory Syndrome Coronavirus 2 (SARS-CoV-2), as well as bacteria such as *Chlamydia pneumoniae* (CP), *Helicobacter pylori* (HP), *Porphyromonas gingivalis* (*P. gingivalis*), Spirochetes and eukaryotic unicellular parasites (e.g., *Toxoplasma gondii*), have been linked to AD due to their ability to activate the immune system, induce inflammation and increase oxidative stress, thereby leading to cognitive decline and AD. In addition, microRNAs (miRNAs) might play a crucial role in the pathogenesis mechanisms of these pathogens since they are utilized to target various protein-coding genes, allowing for immune evasion, maintaining latency, and suppressing cellular signaling molecules. Also, they can regulate gene expression in human cells. This article provides an overview of the association between AD and various infectious agents, with a focus on the mechanisms by which these pathogens may be related to the pathogenesis of AD. These findings suggest important areas for further research to be explored in future studies.

## Introduction

Alzheimer’s disease (AD), the most common type of dementia, especially among the elderly, is recognized as an inflammatory, chronic and progressive neurodegenerative disease (Agostini et al., 2019; Agostini et al., 2017; Bourgade et al., 2016). It is the most prevalent neurodegenerative disease globally, with current estimates of around 24 million affected individuals and projections indicating that this number may increase fourfold by 2050 (dos Santos Picanco et al., 2018). There is ample evidence that AD has been associated with various risk factors, including aging, genetic factors, infectious agents, and environmental factors. However, the underlying etiology of pathological alterations in AD is still not known (Breijyeh and Karaman, 2020).

Senile plaques composed of insoluble amyloid-β (Aβ) peptide and intraneuronal neurofibrillary tangles (NFTs) compopsed of tau protein are the two main pathological characteristics observed in AD brains (Kumar et al., 2015). Notably, Aβ plaques and NFTs are not exclusive to AD; other central nervous system (CNS) disorders, such as chronic infections, also exhibit these specific histopathological markers (Mawanda and Wallace, 2013). The antimicrobial activity of Aβ has been indicated, suggesting that infections may induce the production and deposition of Aβ in the brain (Bourgade et al., 2016; White et al., 2014). Immune response and inflammation are critical components of AD pathogenesis, and an inappropriate immune response in the brain can lead to neurodegenerative processes (Heneka et al., 2014). Increasing Aβ deposits activate glial cells, lymphocytes, and macrophages, which release inflammatory mediators and reactive oxygen species (ROS) (Li et al., 2014). Reactive microglia and astrocytes induce neuronal apoptosis and blood–brain barrier (BBB) dysfunction, leading to the recruitment of peripheral blood leukocytes and exacerbating other AD pathologies (Heneka et al., 2014; Lim et al., 2015; Jacobs and Tavitian, 2012).

The exact mechanism that initiates the upregulation of Aβ aggregation remains mostly unknown. However, the presence of microbial pathogens in brain samples from AD patients indicates that Aβ aggregation may act as an innate immune response to microbial infections (Prosswimmer et al., 2024). Because of their structural similarities, it is proposed that Aβ peptides function as antimicrobial peptides within the innate immune system. Under certain conditions, both antimicrobial peptides and Aβ peptides form *α*-helical structures in the membranes of pathogens, creating ion channels that disrupt cellular homeostasis and lead to cell death (Spitzer et al., 2016). Aβ peptides can self-assemble into Aβ structures, a characteristic often seen in misfolded pathological proteins. These peptides can form channel-like structures in cellular plasma membranes, similar to channel-forming toxins. As a result, the creation of these leaky channels or pores causes the lysis of the targeted organism, ultimately leading to cell death (Bourgade et al., 2016). Moreover, innate immunity against virus infection is impaired in AD, and even in healthy young persons, the immune system cannot completely eradicate pathogens. Repeated activation and latency cycles with infective agent persistence may accelerate immune system senescence. Regulatory mechanisms of innate immunity genes in response to amyloid-Aβ peptide expression have been poorly explored, but it may function as an emergency defense mechanism to compensate for other immune defensive gene inefficiencies in the aging brain (Romagnoli et al., 2020).

The interaction of various infectious agents with environmental, inflammatory, and genetic factors may work as triggers initiating the processes causing Aβ formation, abnormal tau phosphorylation, and consequent neuronal loss (Agostini et al., 2017). Consequently, some possible associations between AD and some infectious agents were investigated, such as Herpes simplex virus 1 (HSV-1) (Bourgade et al., 2015), Epstein Barr virus (EBV) (Ou et al., 2020), Cytomegalovirus (CMV) (Lövheim et al., 2018), Influenza viruses (Jang et al., 2009), Severe Acute Respiratory Syndrome Coronavirus 2 (SARS-CoV-2) (Ciaccio et al., 2021), *Chlamydia pneumoniae* (CP) (Chacko et al., 2022), *Porphyromonas gingivalis (P. gingivalis)* (Gaur and Agnihotri, 2015), Spirochetes (Miklossy, 2008), and *Toxoplasma gondii (T. gondii)* (Nayeri Chegeni et al., 2019).

Mechanisms by which these pathogens may cause AD include the induction of Aβ accumulation (Bourgade et al., 2022; Yirün et al., 2023), tau phosphorylation (Dezfulian, 2018), inflammation (Carbone et al., 2014; Shim et al., 2017), DNA damage, neuronal cell death (Sait et al., 2021), microglial overactivation, reduced brain plasticity (Sadasivan et al., 2015), and impaired Aβ clearance (Liu et al., 2017). Pathogens can penetrate the CNS and stimulate the production of Aβ plaques, neurofibrillary tangles, and tau pathology (Dominy et al., 2019). They can also activate the processing of amyloid precursor proteins (APPs), leading to the progression of AD (Griffin and Barger, 2010). Additionally, inflammatory cytokines and neuroinflammatory markers may contribute to AD pathogenesis (Dezfulian, 2018).

Regarding the role of microRNAs (miRNAs) in the pathogenesis mechanisms, EBV uses miRNAs to target various protein-coding genes, allowing for immune evasion (Lung et al., 2009) and maintaining latency in EBV-associated tumors (Jung et al., 2014). On the other hand, the impact of SARS-CoV-2 on host miRNA populations is relatively minimal (Pawlica et al., 2021); however, the virus does express an miRNA-like small RNA, CoV2-miR-O7a, which is functional in repressing human mRNAs to evade the host immune response (Pawlica et al., 2021). Also, the pathogenesis of *Chlamydia psittaci* and *P. gingivalis* involve the regulation of miRNAs in human bronchial epithelial cells and human periodontal ligament cells, respectively (Chen et al., 2023; Fan et al., 2023), highlighting the importance of miRNA synthesis in understanding viral and bacterial pathogenesis.

In this review article, we aim to give an overview of the association between AD and the infectious agents SARS-CoV-2, HSV-1, CMV, EBV, Influenza viruses, *T. gondii*, HP, Spirochetes, CP, and *P. gingivalis*, focusing on the mechanisms by which these pathogens are related to the AD pathogenesis.

## Viruses

### Herpes simplex virus-1 (HSV-1)

It is a well-established fact that pathogens are involved in AD development, along with HSV-1 gaining some intense attention as a possible risk factor. HSV-1 infection can accelerate the development of AD and similar neurodegeneration by promoting amyloid buildup and neuroinflammation (Berger and Houff, 2008; Kristen et al., 2015; Zhang et al., 2021). Also, the presence of anti-HSV IgM antibodies in the serum, a sign of HSV reactivation, has been associated with an elevated risk of developing AD (Letenneur et al., 2008; Santana et al., 2013). Neuroinflammation plays a critical role in the pathogenesis of AD. The term “neuroinflammation” was introduced to describe an inflammatory response that occurs in the CNS following an injury or infection (Morales et al., 2014). It is important to note that the immune and inflammatory reactions in the CNS differ from those in the rest of the body because of the BBB, which restricts the entry of leukocytes into the brain tissue. Moreover, most of the immune and inflammatory responses in the CNS are driven by the interactions of microglia and astrocytes (Ransohoff et al., 2015). Despite the protective role of neuroinflammation in response to CNS injury or infection, an inappropriate response can lead to neurodegenerative diseases such as AD (Ransohoff, 2016). Pathogens like HSV-1 can activate microglia and astrocytes, leading to the production of inflammatory mediators such as cytokines and chemokines (Hong et al., 2018). This chronic inflammation can damage neurons and promote the accumulation of Aβ plaques, a hallmark of AD (Hong et al., 2018). Studies have shown that HSV-1 infection can induce the recruitment of microglia to the viral core, triggering microglial phagocytosis of HSV-green fluorescent protein (GFP)-positive neuronal cells. This process activates the Nod-like receptor protein 3 (NLRP3) inflammasome pathway, which plays a crucial role in Aβ deposition and the progression of AD (Wang et al., 2024).

HSV-1 typically starts by infecting epithelial cells at mucosal surfaces, such as the mouth or nose. The virus can travel along peripheral nerves to reach the CNS. It often uses the trigeminal nerve or the olfactory nerve to gain access to the brain (Bello-Morales et al., 2020). HSV-1 moves through neurons via retrograde axonal transport, a process where the virus travels backward along the nerve fibers to reach the neuronal cell bodies in the brain (Bello-Morales et al., 2020). Moreover, HSV-1 infection can alter the integrity and permeability of the BBB, allowing other infectious agents to enter the brain parenchyma and exacerbate infection and inflammation. It can lead to the downregulation of tight junction proteins (like occludin and claudin-5) that maintain the tight seal of BBB. This disruption increases the permeability of the BBB, allowing the virus and immune cells to enter the brain (He et al., 2020; Liu et al., 2019). This can lead to a cascade of events that accelerate neurodegeneration and cognitive decline (Feng et al., 2023).

In 1982, Ball suggested a correlation between HSV-1 and AD by observing that herpes simplex encephalitis and AD both impact identical brain regions, and individuals who recovered from herpes simplex encephalitis showed symptoms such as cognitive impairment and memory loss, which are also visible in AD (Ball et al., 1982). AD predominantly affects individuals aged 60 and older, while infections like Herpes and COVID-19 can occur at any age. Despite the difference in age distribution, infections and AD may still be related, as infections can contribute to the development and progression of AD. Many infectious agents, including Herpes viruses, can remain latent in the body and reactivate later in life, particularly under conditions of weakened immunity which are more common in older adults (Oh et al., 2019). This reactivation may then contribute to the pathophysiology of AD. Morover, even if infections occur earlier in life, the long-term inflammatory and immune responses they trigger can have lasting effects on brain health, potentially contributing to neurodegenerative diseases like AD later in life (Sekino et al., 2022). Moreover, about two thirds of persons diagnosed with AD dementia are women (Mielke, 2018). The higher prevalence of AD in women could be influenced by several factors, including the role of pathogens in the pathogenesis of AD. Women generally have a stronger immune response compared to men, which can be a double-edged sword. While a robust immune system can help fight off infections, it can also lead to chronic inflammation, which is a known risk factor for AD (Jung and Mook-Jung, 2024). Additionally, hormonal changes, particularly during menopause, can affect brain health. Estrogen has been shown to have a protective effect on the brain, and its decline during menopause may increase vulnerability to infections and neurodegenerative diseases (Jung and Mook-Jung, 2024). Furthermore, women may experience reactivation of latent infections more frequently due to hormonal fluctuations, which can contribute to chronic inflammation and neurodegeneration (Jung and Mook-Jung, 2024; Filon et al., 2016). Also, it has been noted that there is a high prevalence of certain infectious agents, such as HSV-1, yet a relatively lower incidence of AD. However, it is important to note that not all individuals exposed to these pathogens will develop AD. This may be due to genetic factors, such as the APOE ε4 allele, which is a well-known genetic risk factor for AD. Individuals carrying this allele may be more susceptible to the neurodegenerative effects of infectious agents (Corder et al., 1993). The timing and duration of infection can also play a significant role. Early-life infections or chronic, persistent infections may have a more substantial impact on brain health compared to acute, short-term infections. Moreover, factors such as diet, exercise, exposure to toxins, smoking, and alcohol consumption can influence the risk of developing AD. These factors can interact with infectious agents, either exacerbating or mitigating their effects on brain health (Livingston et al., 2017).

From the overall perspective, the mesial temporal and sub-frontal regions injured through acute Herpes encephalitis are among the regions innervated by trigeminal ganglia-derived fibers. These limbic areas play a crucial role in the recall and memory processes. HSV resides in the trigeminal ganglia of humans and can cause a long-term infiltration of lymphocytes without inducing pathological alterations in those neurons. As a result, these lymphocytes are considered a histological sign of latent Herpes infection whose reactivation can lead to degenerative lesions seen in AD and normal aged brain tissue (Ball et al., 2001).

Table 1 presents an overview of the underlying mechanisms mechanisms that by which HSV-1 is involved in the pathogenesis of AD. There are two pathways through which HSV-1 causes impairments leading to acute neurodegeneration, the APP proteolysis and the autophagy process. An abortive autophagic reaction is promoted by HSV-1, which helps in the accumulation of autophagosomes (Santana et al., 2012). Failure in the degradation of Aβ through autophagy, as well as inhibited secretion of Aβ, can justify the effects of HSV-1 infection on Aβ deposition in autophagic compartments within cells (Santana et al., 2012). It is evident that human herpesvirus 6 (HHV6A) and HSV1, among the neurotropic herpesviruses can affect many kinds of cells existing in the CNS and impair the mechanism of autophagy, which is needed for cellular homeostasis, particularly that of neurons (Tallóczy et al., 2006). To be precise, autophagosome accumulation, which demonstrates an imbalance between autophagosome formation and destruction, has been witnessed in AD patients, and this accumulation may contribute to the extracellular deposition of Aβ and intracellular alterations of tau protein.

**Table 1:** A summary of the mechanisms underlying pathogens involvement in the pathogenesis of Alzheimer’s disease.

Pathogen	Mechanisms	Influence on amyloid deposits and related brain areas
HSV-1	HSV-1 causes impairments through two pathways: APP proteolysis and autophagy process. Inhibited secretion and failure in degradation of Aβ through autophagy are caused by HSV-1 infection leading to Aβ deposition in autophagic compartments within cells. Autophagosome accumulation leads to imbalance between formation and destruction and contributes to Aβ accumulation and tau protein alterations. Attachment of HSV-1 to the plasma membrane of neurons leads to the generation of intra-neuronal Aβ and APP metabolism through an electrophysiological pathway. Ca^2+^-dependent Aβ accumulation facilitated by HSV-1 unbalances intracellular Ca^2+^ homeostasis and produces a self-sustaining vicious circle.	HSV-1 infection can accelerate Aβ deposition by modulating microglial phagocytosis and activating the NLRP3 inflammasome pathway. HSV-1 infection is associated with higher Aβ load in several brain regions, particularly the fronto-temporal regions and the anterior cingulate cortex.
EBV	EBV infection can reduce the biogenesis of mitochondria in monocytes and reduce ROS production and prevent autophagy in infected monocytes and the inflammation caused by reduced autophagy is associated with the pathogenesis of AD. EBV causes neuroinflammation and neuronal loss by infecting peripheral blood mononuclear cells and brain monocytes/macrophages and crossing BBB. TNF-α is highly expressed in the lymphoblastoid cell line that EBV immortalizes of B-cells and leads to aggregation of amyloid β-protein and hyperphosphorylation of tau protein, promoting the development of AD in individuals with AD. EBV-encoded protein BNLF-2a obstructs the transporter associated with antigen processing (TAP), eventually causing the development of AD.	EBV infection can exacerbate neuroinflammation and oxidative stress, further promoting the aggregation of Aβ and tau proteins. EBV-related Aβ deposits are often found in the temporal lobe, particularly in the hippocampus and entorhinal cortex.
CMV	CMV has been associated with downregulation of cell-mediated immunity, resulting in increased cellular and inflammatory markers commonly linked to cognitive decline. CMV-specific CD8^+^ T cells can generate interferon γ, and there is a significant correlation between increased levels of CMV IgG antibodies and higher levels of TNF-α and IL-6 in older adults. This immune and inflammatory pathway linked to CMV is related to cognitive decline and AD.	CMV infection can lead to a persistent immune response, causing chronic inflammation in the brain. This inflammation can activate microglia and astrocytes, which are involved in the production and deposition of Aβ. The infection triggers the release of pro-inflammatory cytokines, which can exacerbate neuroinflammation and promote Aβ accumulation. CMV can compromise the integrity of the blood–brain barrier, allowing immune cells and inflammatory molecules to enter the brain more easily. This disruption can facilitate the deposition of Aβ in the brain. MV can express viral proteins that interfere with normal cellular processes, potentially increasing the production of Aβ precursor protein (APP) and its cleavage into Aβ peptides. The presence of CMV can increase oxidative stress in neurons, which is known to promote the aggregation of Aβ and tau proteins CMV-related Aβ deposits are often found in the temporal lobe, particularly in the hippocampus and entorhinal cortex. The frontal cortex can also be affected by CMV-induced inflammation and Aβ deposition.
Influenza virus	Aβ42 has been found to aggregate influenza virus, attract neutrophils, and enhance hydrogen peroxide release by neutrophils, suggesting it may have antiviral properties. CA/09 H1N1 may cause overactivation of microglia and lower expression levels of BDNF and GDNF, increasing the risk of AD. influenza type A virus infection can activate inositol requiring enzyme 1 (IRE1) causing X-box binding protein 1 (XBP1) splicing and inducing ER stress, which is expected to be involved in AD pathogenesis.	Influenza virus contributes to the pathogenesis of AD by promoting chronic neuroinflammation, disrupting the BBB, and inducing oxidative stress. These mechanisms collectively lead to increased Aβ deposition. Influenza virus-related neuroinflammation and Aβ deposition are often observed in the hippocampus. The prefrontal cortex can also be affected by influenza-induced inflammation and Aβ deposition.
SARS-CoV-2	Neuroinflammatory responses and BBB disruption result in the penetration of infected lymphocytes and monocytes to the CNS, activating microglia and astrocytes and causing elevated ROS production and subsequent damage to synapses and neurons. High levels of cytokines can affect hippocampal atrophy, which is a common feature among AD patients and correlated with cognitive impairment. Aβ production is augmented as an immune response to SARS-CoV-2, resulting in Aβ deposition and excess of protein accumulation in senile plaques, particularly in the hippocampus, which is the primary pathophysiological mechanism that causes AD. Gut microbiota dysbiosis may play a significant role in the spread of the virus and its invasion into the CNS, impacting susceptibility to infectious and inflammatory diseases like COVID-19 and neurodegenerative disorders like AD. ACE-2 depletion caused by SARS-CoV-2 infection can increase the risk of neurodegenerative diseases.	SARS-CoV-2 infection can trigger a severe immune response known as a cytokine storm, leading to widespread inflammation in the brain. This inflammation activates microglia and astrocytes, which can increase Aβ production and deposition. The infection induces oxidative stress, which can further promote the aggregation of Aβ and tau proteins. SARS-CoV-2 proteins may directly interact with neuronal cells, potentially influencing the production and aggregation of Aβ. SARS-CoV-2-related neuroinflammation and Aβ deposition are often observed in the hippocampus. The frontal cortex can also be affected.
CP	Transmigration of infected monocytes into brain activates astrocytes and glial cells, releasing cytokines that trigger BACE and γ-secretase to produce Aβ. CP can reach CNS through olfactory route by infecting neuroepithelium, reaching olfactory bulb, entorhinal cortex, hippocampus, and temporal cortex, and inducing inflammation in glial cells, leading to AD. Phage DNA transcribes into miRNAs that interfere with human mRNAs, infect mitochondria, cause mutation in mtDNA and OXPHOS enzymes, inhibit ATP production, aggregate dysfunctional proteins, produce chaperones, upregulate immune cells, and activate mtPTP to induce apoptosis in brain cells and cause AD.	CP infection activates microglia. Activated microglia release pro-inflammatory cytokines such as IL-1β, TNF-α, and IL-6. These cytokines can enhance the production of Aβ by increasing the activity of enzymes like beta-secretase (BACE1) and gamma-secretase, which cleave APP into Aβ peptides. CP pneumoniae can infect neurons and glial cells directly. This infection can alter cellular processes, leading to increased APP expression and its subsequent cleavage into Aβ. CP infection in frontal cortex can lead to significant cognitive impairments due to increased Aβ deposition and inflammation. The temporal cortex, including the hippocampus, can also be affected.
HP	NMR of HP-infected cells reveals excessive levels of BCAA isoleucine, leucine, and valine altering BCAA and glutamate metabolism, potentially alleviating AD risk. Elevated BCAA plasma levels and inhibited tryptophan in the brain influence serotonin production, Aβ formation, and neural survival. HP induces over-activation of mTORC1, causing BBB breakdown through endothelial cell dysfunction, tau hyperphosphorylation, and Aβ aggregation, due to inhibition of autophagy. HP releases acids that disturb microglial and astrocyte function, contributing to Aβ and tau phosphorylation and AD progression. HP-n can cross BBB through LRP-1 and RAGE, potentially causing AD through Aβ plaques.	*H. pylori* infection can lead to systemic inflammation, which may extend to the brain. This inflammation activates microglia and astrocytes, increasing the production and deposition of Aβ. *H. pylori* produces toxins such as urease, which can induce oxidative stress and inflammation in neurons, promoting Aβ aggregation. *H. pylori* can compromise the integrity of the blood–brain barrier, allowing immune cells and inflammatory molecules to enter the brain more easily. This disruption facilitates the deposition of Aβ in the brain. *H. pylori*-related neuroinflammation and Aβ deposition are often observed in the hippocampus. The frontal cortex can also be affected.
*P. gingivalis*	Periodontitis caused by *P. gingivalis* leads to chronic inflammation, tooth loss, and systemic inflammatory response, and contributes to AD. Gingipains start cleavage of pro-caspase-3, producing caspase-3 associated with enhanced tau phosphorylation, leading to impaired neuronal function and Aβ formation and neural cell death. *P. gingivalis* OMVs induce NLRP3 inflammasome activation and cause Aβ formation, leading to pre-apoptosis and neural cell death. *P. gingivalis* LPS preparations stimulate GSK-3b activation in microglia, leading to tau hyperphosphorylation and *APP* expression and downstream neuroinflammation. *P. gingivalis*-induced systemic inflammation may contribute to endothelial dysfunction and overexpression of platelet aggregation proteins and atherosclerosis, contributing to AD. *P. gingivalis* infection impairs the sleep patterns and circadian system, leading to decreased clearance of Aβ peptides and an elevation in accumulation of aggregated proteins.	*P. gingivalis* infection can lead to systemic and brain inflammation. This inflammation activates microglia and astrocytes, increasing the production and deposition of Aβ. The infection triggers the release of pro-inflammatory cytokines, exacerbating neuroinflammation and promoting Aβ accumulation. *P. gingivalis* produces enzymes called gingipains, which can degrade neural proteins and disrupt normal brain function. These enzymes can also interact with APP, increasing Aβ production. The frontal cortex and hippocamous are mainly affected by *P. gingivalis*-induced inflammation and Aβ deposition
Spirochetes	Osp A protein is an amyloid executor leading to Aβ formation. TLR2 induces the release of TNF-α and NF-κB through MYD88, which activate *α*-, β-, and γ-secretase, leading to Aβ production and destruction of surrounding tissue. Iron aggregation leads to reactive oxygen and oxidative stress, inducing pro-inflammatory cytokines and Aβ deposition.	The infection triggers the release of pro-inflammatory cytokines, exacerbating neuroinflammation and promoting Aβ accumulation. Spirochete-related Aβ deposits are often found in the cortex, including the frontal and temporal lobes. The hippocampus is also affected.
*T. gondii*	*T. gondii* induces immune responses and inflammation in the CNS, alters neurotransmitter levels, and activates indoleamine-2,3-dyoxigenase, potentially contributing to AD. Inflammatory responses protect against *T. gondii* but can also damage non-infected neurons and affect neurotransmitter function, stimulating AD progression. *T. gondii* increases the secretion of IFN-δ and NO, leading to neural degeneration and AD progression. *T. gondii* disturbs NMDAR signaling, impacting Aβ accumulation and hyperphosphorylation of tau, contributing to AD. Infection with *T. gondii* has both pro- and anti-inflammatory effects, and more research is needed to understand fully its role in AD pathogenesis.	Chronic *T. gondii* infection enhances the recruitment of monocytes to the brain. These monocytes have a high capacity for phagocytosing Aβ, which can reduce Aβ plaque load. The infection increases the expression of enzymes involved in the degradation of Aβ, such as insulin-degrading enzyme (IDE) and matrix metalloproteinases (MMPs). *T. gondii* infection has been shown to reduce amyloid burden in various cortical regions, including the prelimbic cortex, retrosplenial cortex, and visual cortex. The hippocampus also shows reduced Aβ deposition following *T. gondii* infection.

AD: Alzheimer’s disease; HSV-1, Herpes simplex virus-1; EBV, Epstein–Barr virus; CMV, Cytomegalovirus; CP, Chlamydia pneumonia; HP, *Helicobacter pylori*; *P. gingivalis*, *Porphyromonas gingivalis*; *T. gondii*, *Toxoplasma gondii*; APP, amyloid precursor protein; Aβ, amyloid-β; ROS, reactive oxygen species; TNF-α, tumor necrosis factor -α; BDNF, brain-derived neurotrophic factor; GDNF, glial cell-derived neurotrophic factor; CNS, central nervous system; ACE-2, angiotensin-converting enzyme-2; BASE, Aβ converting enzyme; OMVs, outer membrane vesicles; NMDAR, N-methyl-D-aspartate receptor.

Furthermore, herpesviral infection of microglial and other glial cells can elevate the generation of ROS by promoting inflammation and mitochondrial dynamic changes, which can be considered as another AD sign. HSV-1 has also been demonstrated to cause the accumulation of Aβ42 in neuronal cells and human induced pluripotent stem cells from healthy individuals, even at low infection levels (Abrahamson et al., 2021; Wozniak et al., 2011). In addition, studies have revealed that rat cortical neurons infected with HSV-1 showed higher levels of intracellular Ca^2+^, which triggered the Ca^2+^-dependent phosphorylation of APP and the subsequent intracellular accumulation of Aβ42 (Piacentini et al., 2011). So, a significant alteration will occur in the homeostasis and intracellular Ca^2+^ ([Ca^2+^]i) signaling of the neural cells infected with HSV-1. Also, HSV-1 infection induces intracellular Ca2+ transients that generate a perceivable elevation in basal [Ca^2+^]i within a few minutes. It is worth mentioning that HSV-1-induced elevation in [Ca^2+^]i has also been detected in cervical cancer and renal epithelial cells. They are mostly caused by inositol 1,4,5-trisphosphate receptor (IP3R) activation, which leads to Ca^2+^ discharge from the endoplasmic reticulum (ER). The Ca^2+^ signaling in these cells is conceivably assigned to the relationship between heparan sulfate proteoglycans existing on the cell membrane and viral glycoproteins gC and gB, which prompt G-protein-dependent stimulation of the phospholipase C *γ* that hydrolyzes phosphatidylinositol 4,5-bisphosphate to IP3. Attachment of HSV-1 to specific receptors, such as nectin-1 (HveC, CD111) and heparan sulfate (Karasneh and Shukla, 2011), on the plasma membrane of neurons activates a pathway of electrophysiological reactions, leading to the generation of intra-neuronal Aβ and altered APP metabolism. The alteration of ion channels is an initial event leading to subsequent incidents, allowing the virus to trigger APP phosphorylation. These ion channels function following neuronal firing or at the resting membrane potential. Subsequently, crucial tasks assisted by intracellular Ca^2+^ signals, which are mainly linked to Ca^2+^ influx through VGCCs, discharge calcium from intracellular stores. Besides, the Ca^2+^-dependent Aβ accumulation facilitated by HSV-1 may later unbalance the intracellular Ca^2+^ homeostasis and hence produce a self-sustaining vicious circle (Piacentini et al., 2011).

### Epstein–Barr virus (EBV)

One of the most prevalent herpesviruses, recognized for its asymptomatic infection in most adults, is human herpesvirus 4 (HHV4), also known as EBV (Jha et al., 2015). This double-stranded DNA virus mainly infects B lymphocytes (Jha et al., 2015). Based on the previous studies, EBV may play a role in the pathogenesis of AD (Shim et al., 2017; Zhang et al., 2022). Below is a brief outline of some of the mechanisms which can some mechanisms potentially associated with AD. To begin with, Talwar et al. investigated the interaction between *Hepatitis C virus* (HCV), EBV, *Human Herpes Virus 8* (HHV8), and HPV and AD candidate genes, including *AKT1*, *GSK3B*, *APP*, *APOE*, *EGFR*, *PIN1*, *CASP8*, and synuclein alpha (*SNCA*) (Talwar et al., 2019). In their study, the involvement of EBV with epidermal growth factor receptor (EGFR), which affects cell proliferation, growth, and survival, has been shown (Talwar et al., 2019; Shafi, 2016). Moreover, epidermal growth factor (EGF) is a peptide that regulates neural stem cells and plays a role in neurogenesis in the hippocampus and the improvement of cognitive functions (Shafi, 2016). Furthermore, Thomas et al. demonstrated that EGF prevents impairment of cognitive function and cerebrovascular defects (Thomas et al., 2017). So EGFR levels are one of the parameters that can be used to distinguish AD patients from controls (Talwar et al., 2019). Also, some therapeutic options (e.g., angiogenic growth factors [AGF]-like drugs) may be provided to reduce AD risk (Thomas et al., 2016).

It is widely accepted that EBV utilizes autophagic machinery to increase viral production. EBV can inhibit the last phases of the autophagy process, through the interaction of its protein TRS1 with Beclin1 (Romeo et al., 2020). Reduction of autophagy in infected monocytes leads to the accumulation of p62/SQSTM1 and Nuclear factor erythroid 2-Related Factor 2 (NRF2) up-regulation which prevents escalation in reactive oxygen species (ROS) levels induced by Interleukin-4 (IL-4) and Granulocyte-Macrophage Colony-Stimulating Factor (GM-CSF). On the other hand, EBV infection can reduce the biogenesis of mitochondria in monocytes and by this mechanism, it can reduce ROS production and prevent autophagy in infected monocytes. Reduction of ROS can strongly impair the formation of dendritic cells from monocytes. Also, increased ER stress and the provocation of the unfolded protein response (UPR) play an important role in the inflammation, because of the reduction of autophagy (Romeo et al., 2020). Ultimately, it can be said that this inflammatory process is associated with the pathogenesis of AD (Talwar et al., 2019; Carbone et al., 2014).

Having considered the association of EBV and serological markers, a previous study has pointed out that anti-EBV IgG levels were considerably higher in patients with amnestic mild cognitive impairment (aMCI) in comparison with the control group over the 2-year follow-up period in Korean elderly people (Shim et al., 2017). In addition, EBV causes neuroinflammation and neuronal loss in the brain by infecting peripheral blood mononuclear cells and brain monocytes/macrophages and crossing the BBB (Kanakry et al., 2016). Decreased levels of the cytokine TNF-*α* can reduce the hyperphosphorylation of tau protein (Dezfulian, 2018). In individuals with AD, TNF-α is highly expressed in the lymphoblastoid cell line that EBV immortalizes of B-cells, leading to aggregation of amyloid β-protein and hyperphosphorylation of tau protein, ultimately promoting the development of AD (Dezfulian, 2018). Gate et al. discovered that EBNA3A and BZLF-1 antigens trigger an immune response mediated by CD8^+^T_EMRA_ cells associated with immune memory, which are negatively correlated with cognitive performance. In individuals with AD, CD8^+^T_EMRA_ cells release pro-inflammatory cytokines, such as IFNγ, TNF-α, and cytotoxic factors (NKG7, GZMA, and B2M), which lead to a decline in cognitive function and exacerbate the symptoms of AD (Carbone et al., 2014; Gate et al., 2020; Kang and Liu, 2020; Tiwari et al., 2021). Moreover, EBV-encoded protein BNLF-2a obstructs transporter associated protein (TAP), thus triggering the downregulation of MHC-I and II expression. By doing so, neuronal cells accumulate viral polypeptides in the environment, eventually causing the development of AD (Tiwari et al., 2021). EBV can also trigger a stress immune response that causes inflammation and cognitive decline during aging, both in its latency and reactivation phases (Carbone et al., 2014; Shim et al., 2017). However, there are few studies on how EBV contributes to AD, and more studies are necessary to fully understand the pathogenesis.

miRNAs also play a significant role in the relationship between EBV and AD by regulating gene expression and influencing various cellular processes. They are crucial in regulating inflammatory responses. Dysregulation of specific miRNAs can lead to chronic inflammation, which is a known factor in AD pathogenesis (Sequeira and Godad, 2024). Certain miRNAs are involved in the metabolism of Aβ and tau proteins, both of which are central to AD pathology. For instance, miRNAs can influence the expression of enzymes like BACE1, which is involved in Aβ production (Sequeira and Godad, 2024). Moreover, miRNAs play roles in maintaining synaptic health and plasticity. Dysregulation of these miRNAs can contribute to synaptic dysfunction, a hallmark of AD (Abidin et al., 2023). They are also involved in cellular stress responses, including oxidative stress and autophagy, which are critical in the context of neurodegenerative diseases (Abidin et al., 2023). Furthermore, factors such as diet, pollutants, stress, and lifestyle choices can lead to epigenetic modifications. For example, exposure to pollutants or a poor diet can alter DNA methylation patterns, which may affect genes involved in immune response and inflammation (Klibaner-Schiff et al., 2024; Migliore and Coppedè, 2022). These environmental exposures can induce changes in the epigenome, such as DNA methylation and histone modifications, which can influence the expression of genes related to both EBV and AD. For instance, altered DNA methylation patterns can affect the expression of genes involved in amyloid-β production and tau phosphorylation (Liu et al., 2018; Xiao et al., 2020). Epigenetic changes can mediate the effects of environmental factors on gene expression, potentially exacerbating the impact of EBV on neuroinflammation and neurodegeneration. For example, chronic stress can lead to epigenetic changes that enhance the inflammatory response, which is a known factor in both EBV infection and AD (Migliore and Coppedè, 2022; De Plano et al., 2024). Environmental factors can also affect the expression of miRNAs, which play a crucial role in regulating gene expression. Dysregulated miRNAs can impact the expression of genes involved in immune response, inflammation, and neuronal health, thereby influencing the relationship between EBV and AD (Xiao et al., 2020; De Plano et al., 2024).

Several studies have identified different EBV-encoded miRNAs and their targets. For example, miR-BART21 and miR-BART22 are highly expressed in NPC and modulate the expression of the immunogenic viral antigen LMP2A, allowing escape of EBV-infected cells from host immune surveillance (Lung et al., 2009). Moreover, miR-BART20-5p helps maintain latency in EBV-associated tumors by directly targeting immediate early genes *BZLF1* and *BRLF1* (Jung et al., 2014). Furthermore, EBV encodes miRNAs (e.g., BART 18-5p) that suppress the cellular signaling molecule MAP kinase kinase kinase 2 (MAP3K2) at exactly the same site as the oncogenic cellular miRNA mir–26a-5p, thus blocking viral replication and maintaining latency in memory B cells (Qiu and Thorley-Lawson, 2014). Additionally, EBV-miR-BART2 targets the viral DNA polymerase BALF5, which inhibits the transition from latent to lytic viral replication (Barth et al., 2008). Finally, miR-BART6 of EBV is edited in latently infected cells, suppressing processing of miR-BART6 RNAs and silencing Dicer through multiple target sites located in the 3′-UTR (untranslated region) of Dicer mRNA (Iizasa et al., 2010).

### Cytomegalovirus (CMV)

Cytomegalovirus (CMV), similar to EBV, is from the Herpesviridae group (Lövheim et al., 2018). CMV can be transmitted from one person to another through contact with bodily fluids of persons who have symptomatic or asymptomatic infection (Barnes et al., 2015). Investigations have linked CMV to an increased risk of AD and cognitive decline associated with aging. CMV may also contribute to cognitive decline in elderly individuals and dementia in patients with Down syndrome (Licastro et al., 2011). A study found that high CMV antibody levels were linked to faster cognitive decline over four years (Aiello et al., 2006). Both AD patients and healthy elderly individuals tested positive for CMV, but there was no significant difference between the groups. Moreover, CMV was more frequently found in patients with vascular dementia, suggesting a potential role for the virus in this condition (Carbone et al., 2014). Although some findings suggest that there is no direct correlation between CMV and AD pathogenesis (Ji et al., 2023), several studies revealed an association between CMV infection and AD (Lövheim et al., 2018). Numerous studies have reported that there is a relationship between CMV serological markers and AD. A clear example is the study by Barnes et al., in which the authors showed the association of CMV seropositivity with enhanced the risk of AD development (relative risk, 2.15; 95% confidence interval [CI], 1.42–3.27) in a cohort study with 849 participants (Barnes et al., 2015). In another study, a significant correlation between the CMV seropositivity and AD has been reported by Bu et al. (adjusted odds ratio, 2.33; 95%CI, 1.14–4.77) (Bu et al., 2015). Furthermore, a study carried out by Lurain et al. showed that there is an association between increased levels of anti-CMV IgG and density of NFTs (Lurain et al., 2013). In sum, given above-mentioned studies, there might be a relationship between CMV and AD.

However, the exact mechanisms linking CMV to the risk of AD are unclear. Despite often being undiagnosed due to its asymptomatic nature, CMV remains in a latent state within the immune system, with a higher likelihood of reactivation in older age (Koch et al., 2006). Several factors suggest CMV might be linked to AD risk through its impact on the aging immune system. Firstly, older adults exhibit higher levels of IgG antibodies to CMV compared to younger individuals (Barnes et al., 2015), and aging-related changes in cell-mediated immune parameters can lead to subclinical CMV reactivation (Stowe et al., 2007). Secondly, CMV has been associated with the downregulation of cell-mediated immunity, resulting in increased cellular and inflammatory markers commonly linked to cognitive decline (Koch et al., 2006; Almanzar et al., 2005). CMV-specific CD8^+^ T cells can generate interferon *γ* (Almanzar et al., 2005), and there is a significant correlation between increased levels of CMV IgG antibodies and higher levels of tumor necrosis factor *α* and IL-6 in older adults (Roberts et al., 2010; Schmaltz et al., 2005). This immune and inflammatory pathway that is linked to CMV is also related to cognitive decline and AD (Zhao et al., 2024).

### Influenza viruses

Influenza viruses are single-stranded RNA viruses belonging to the Orthomyxoviridae family (Luo, 2012). Influenza and pneumonia has been significantly linked to five out of six neurodegenerative diseases (AD, ALS, dementia, Parkinson’s disease [PD], and vascular dementia). These associations has been confirmed using cross-sectional data from the UK Biobank (Levine et al., 2023).

However, there is some argument over the role of these viruses in AD. The virus can change through antigenic drift and shift. The latter can cause epidemics like avian flu (H5N1) and swine flu (H1N1), named after the variant proteins that allow the virus to enter host cells and facilitate lysis after replication (Piekut et al., 2022). H5N1 has been associated with the phosphorylation and aggregation of alpha-synuclein, which is known to play a significant role in PD neurodegeneration (Jang et al., 2009). Although this pathology is more evident in Lewy body dementia and PD, the structural resemblance between Aβ and influenza hemagglutinin indicates that H5N1 might also contribute to AD (Piekut et al., 2022). The similarities are particularly notable in the C-termini of both proteins, where hemagglutinin contains a domain responsible for cell membrane binding. This aligns with the hypothesis that influenza virus-induced membrane-poration could lead to neurotoxicity (Piekut et al., 2022). Aβ42 is a significant component of Aβ plaques found in AD, and recent studies suggest that it may have antiviral properties. In one study, Aβ42 was found to aggregate influenza virus, attract neutrophils, and enhance hydrogen peroxide release by neutrophils (White et al., 2018). The ability to aggregate is thought to be caused by a C-terminal loop in Aβ42 that includes residues Met35 to Ala42, which is also involved in Aβ42 oligomerization (Ahmed et al., 2010; Wagoner et al., 2014). Also, H5N1 can penetrate the CNS through the peripheral nervous system and cause innate immune system activation and subsequently dopaminergic neuronal degeneration in the substantia nigra pars compacta (SNpc) (Jang et al., 2009). Although this dopaminergic neurons loss can be restored after about 90 days of infection, a long-term inflammation and activation of microglia can cause some impairments in the neural cells (Limphaibool et al., 2019). So, some disorders that are related to the aggregation of proteins and neurodegeneration can take place due to the activation of microglial cells (Limphaibool et al., 2019; Sadasivan et al., 2015).

CA/09 H1N1 is another strain of influenza virus that may be associated with neurological changes. Although it is not believed to actively cross the BBB and is considered non-neurotropic (Sadasivan et al., 2015), one study showed that it causes overactivation of microglia, which persisted for up to 90 days post-infection (Sadasivan et al., 2015). Infection with this virus also results in a decrease in brain-derived neurotrophic factor (BDNF) and glial cell-derived neurotrophic factor (GDNF), which encode key factors essential for maintaining neural plasticity. BDNF and GDNF are also responsible for regulating microglial activation, and lower expression levels may lead to inflammation in the CNS that, coupled with reduced brain plasticity, increases the risk of AD (Sadasivan et al., 2015).

Ultimately, Hassan et al. revealed that there is a correlation between unfolded protein response (UPR), ER stress, and the pathogenesis of influenza type A virus (IAV) infection. In this study, it has been indicated that inositol requiring enzyme 1 (IRE1) can be activated by IAV infection. Subsequently, activated IRE1 can cause X-box binding protein 1 (XBP1) splicing resulting in modulation of pro-survival responses (Mehrbod et al., 2019). Therefore, it is expected that this mechanism is involved in AD pathogenesis, as chronic ER stress and prolonged UPR activation can lead to neuronal dysfunction and death. Specifically, the persistent activation of IRE1 and XBP1 splicing may contribute to the accumulation of misfolded proteins, such as Aβ and tau, which exacerbate neuroinflammation and oxidative stress, further promoting neurodegeneration (Marques et al., 2024; Marques et al., 2023).

Vaccines can help reduce the risk of various neurodegenerative diseases including AD and PD. Studies have shown that getting vaccinated for influenza and pneumonia can lower the risk of AD, especially if the pneumonia vaccine is given between ages 65–75. Protection from bacterial and viral infections can be helpful for the brain because they may activate dormant viruses such as HSV-1 and HZV that could contribute to AD. The Zostavax vaccine for shingles has also been found to reduce the risk of AD and PD, supporting the idea that viruses may play a role in neurodegeneration (Lehrer and Rheinstein, 2022). Observational studies and a meta-analysis have shown evidence that influenza vaccination may be linked to a lower risk of dementia (Veronese et al., 2022; Liu et al., 2016; Luo et al., 2020). A large cohort study involving over 2 million participants also reported a 40% reduced risk of AD among vaccinated elderly individuals (Bukhbinder et al., 2022). Experiments in mice have shown that flu vaccination affects microglial activity and Aβ clearance (Yang et al., 2020), supporting the theory that the protective impact of flu vaccination on dementia may be due to nonspecific effects on the immune system (Hjelholt et al., 2023).

In brief, foregoing discussions indicates that mechanisms mentioned above have profound impact in the AD pathogenesis by activating immune system, aggregating proteins, and inducing ER stress.

### SARS-CoV-2

In December 2019, a coronavirus, SARS-CoV-2, emerged in China, which can cause severe acute respiratory syndrome (SARS). COVID-19 pandemic dramatically impacted people’s lives worldwide in various ways, including health, economic, political, and social (Nicola et al., 2020). Although currently, to minimize the mortality rate, it is mainly focused on the relief of cardiovascular and pulmonary consequences of COVID-19, there are also reports of neurological presentations in the cases (Mao et al., 2020; Vakili et al., 2021). This virus, like other human coronaviruses, is considered to be an opportunistic microorganism of the CNS (Desforges et al., 2020). According to post-mortem investigations, the SARS-CoV-2 antigen and RNA were found in the brains of the patients (Matschke et al., 2020). Therefore, the assumption has been raised that SARS-CoV-2 infection may lead to long-term neurological consequences in particular cognitive decline and dementia, which draw our attention to AD (Mao et al., 2020). The findings of Baranova et al. suggest that COVID-19 infection may contribute to the development of clinical AD. The study reveals a positive genetic correlation between hospitalized COVID-19 and AD, with genetic liabilities to severe COVID-19 associated with an increased risk for the latter (Baranova et al., 2023). Moreover, it has been shown that Covid-19 infection is linked to an increase in the development of new onset clinical AD. Wang et al. that those who had contracted Covid-19 were at a significantly higher risk of being newly diagnosed with AD within a 360-day period after their initial diagnosis of Covid-19 (with a hazard ratio of 1.69 and a 95% confidence interval ranging from 1.53 to 1.72) (Wang et al., 2022). Interestingly, despite the difference in age distribution between Covid-19 and AD, they may still be associated. While Covid-19 has the potential to affect individuals of all ages, older adults are more susceptible to severe infections and complications that can have neurological impacts. Also, the processes by which Covid-19 contribute to AD may interact with aging mechanisms. For example, age-related decline in immune function (immunosenescence) could worsen the effects of infections on the brain.

It has been demonstrated that it is the interaction between the S1 spike protein of SARS-CoV-2 and angiotensin-converting enzyme-2 (ACE-2) which is responsible for the invasion of this virus to the cells and ACE-2 is highly represented in brain tissue as well (Alberti et al., 2009; Dolatshahi et al., 2021). In brain, ACE-2 is expressed on neurons, glial cells, endothelial cells, smooth muscle cells of arteries, and also hippocampus and temporal lobe involved in AD pathogenesis (Dong et al., 2020).

A number of interrelated pathways are usually observed which led to SARS-CoV-2 entrance into CNS. first and foremost, due to the virus interaction with ACE-2 receptors on the endothelium, BBB is disrupted. Thus, the infected lymphocytes can cross the barrier (Varga et al., 2020; Hascup and Hascup, 2020). Another route is the axonal transport of the virus through olfactory neurons (Mao et al., 2020). Transmembrane serine protease 2 (TRPMSS2), and ACE-2 are abundantly expressed on the olfactory epithelium. After the infection, TRPMSS2 expression is elevated in olfactory neurons and facilitates virus entrance. Also, changes in olfaction are a primary symptom of neurodegenerative diseases including AD (Bagasra et al., n.d.). On the other hand, lymphatics or hematogenous dissemination is another probable pathway (Bostancıklıoğlu, 2020). Virus receptors, including ACE-2, TMPRSS2, and FURIN, are expressed by dorsal root ganglion (DRG) and sensory neurons (Shiers et al., 2020). Therefore, free nerve terminals of skin or lumens epithelium are another potential route that contributes to the virus’s entry into the brain. Furthermore, one of the determining pathways which could account for the transmission of virus into the brain is the vagus nerve and enteric nervous system (Esposito et al., 2020). P2X7 receptors are known as ion channels expressed in the CNS which can be triggered by ATP originating from distressed cells (Ribeiro et al., 2021; Di Virgilio et al., 2017; Sluyter, 2017). SARS-CoV-2 augments extracellular ATP, which can stimulate P2X7 receptors hyperactivation. This, in turn, triggers NOD-like receptor protein 3 (NLRP3) inflammasome (Ribeiro et al., 2021). Other factors responsible for NLRP3 activation are the open reading frame 3a (ORF3a) protein of the virus, systemic inflammation, and acute lung injury. Subsequently, NLRP3 activation upregulates cytokines such as interleukin-1beta (IL-1β), increases pathogenic fibrils by increasing aggregation of peptides, causes mitochondrial failure, apoptosis, and thus neurodegeneration, which is a hallmark of AD (Siu et al., 2019; Heneka et al., 2013). Inflammatory mediators such as IL-1β, 2 and 6 and TNF-*α* are elevated in COVID-19 cases and assist in this CNS alterations. Moreover, Stain et al. found that SARS-CoV-2 is present in various tissues, including the brain, during early stages of infection, especially in severe COVID-19 patients who died. Their study also demonstrated that SARS-CoV-2 RNA can persist in multiple sites, even in the brain up to 230 days after the onset of symptoms in one case (Stein et al., 2022).

**Figure 1:**
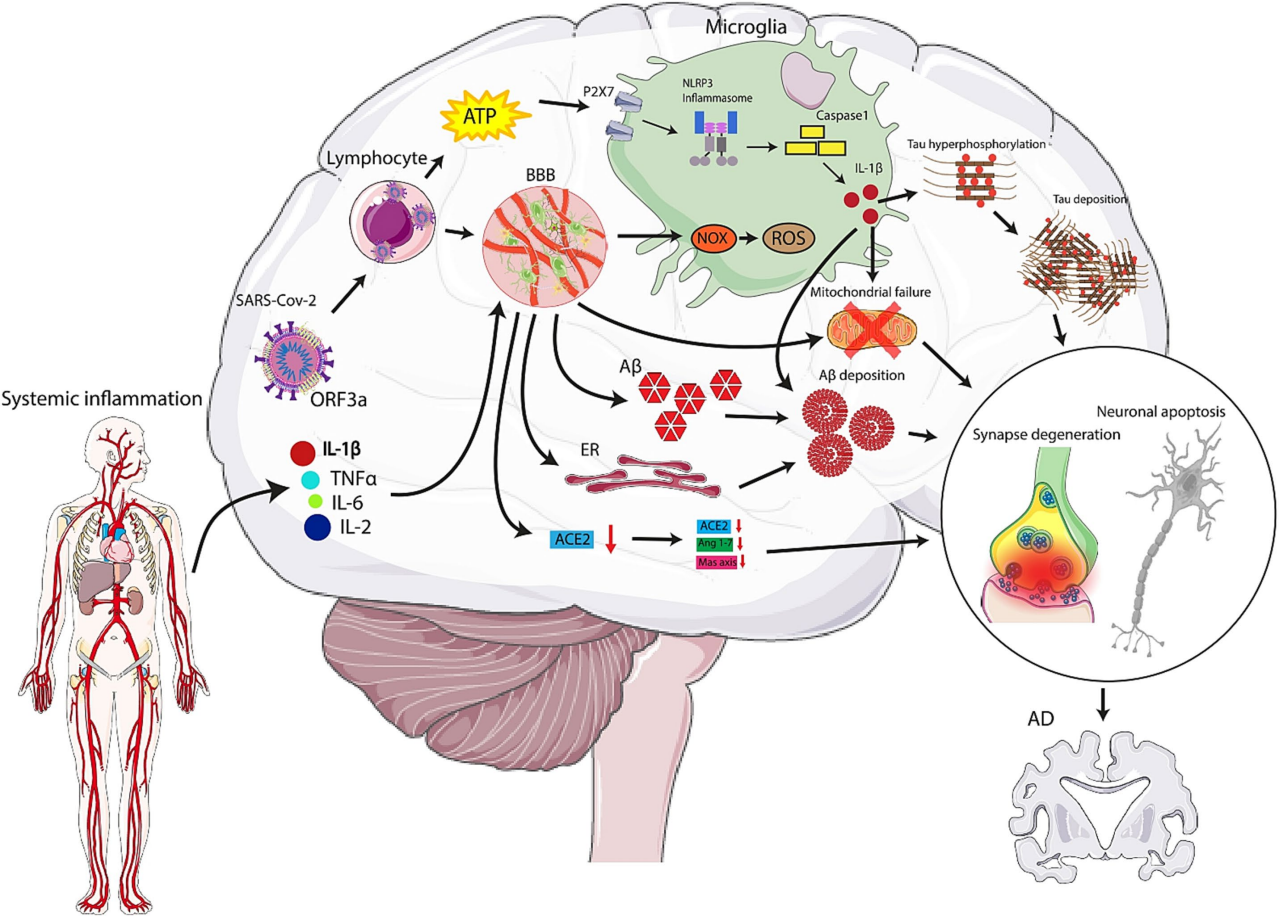
Mechanisms by which SARS-CoV-2 can play role in the pathogenesis of Alzheimer’s Disease (AD). High levels of cytokines and chemokines can cross the blood brain barrier (BBB), causing neuroinflammatory responses and disruption. Virus-infected lymphocytes and monocytes penetrate the central nervous system (CNS), leading to activation of microglia and astrocytes and resulting in neuroinflammation. Activated microglia trigger NADPH oxidase (NOX) enzyme, significantly increasing reactive oxygen species (ROS) production, which impacts neuronal oxidative disruption and can aggravate neurodegenerative diseases, including AD. Also, the virus can enter the nasal epithelium and travel along the olfactory nerve fibers. It uses the ACE2 receptor, present in the nasal epithelium, to enter cells. From there, it hijacks the cellular machinery to replicate and spread along the olfactory nerve. Once the virus reaches the olfactory bulb, it can potentially spread to other parts of the brain. The proximity of the olfactory bulb to the brain allows the virus to access deeper brain structures.

Above all, high levels of cytokines and chemokines can cross BBB, resulting in neuroinflammatory responses and BBB disruption. Thus, lymphocytes and monocytes infected by the virus, penetrate to CNS and lead to activation of microglia and astrocytes and thus neuroinflammation (Chaves Filho et al., 2021; Jakhmola et al., 2020; Yarlagadda et al., 2009). It is the activated microglia by which nicotinamide adenine dinucleotide phosphate (NADPH) oxidase (NOX) enzyme is triggered and therefore elevates ROS production significantly. ROS impact, not only neuronal oxidative disruption, but also the development or aggravation of neurodegenerative diseases (Cahill-Smith and Li, 2014; Geng et al., 2020). It should be stated that NOX2 plays a role in the pathogenesis of neurodegenerative disorders, including AD (Kumar et al., 2016). Recently Violi et al. have attempted to assess the correlation between COVID-19 clinical exacerbation and NOX2 levels (Violi et al., 2020). It is proven that the continuous activation of microglia contributes to the activation of other microglia and causes elevated tau hyperphosphorylation, mitochondrial failure, and apoptosis. This causes damage to both synapses and neurons, which ultimately contributes to neurodegeneration (Miksys and Tyndale, 2006; Domingues et al., 2017). Ultimately, It has been proven that systemic inflammation, specifically high levels of cytokines, similar to sepsis, can affect hippocampal atrophy (Lindlau et al., 2015; Heneka et al., 2020), which is a common feature among AD patients, and correlated with cognitive impairment (Lu et al., 2020) (Figure 1).

Another mechanism explaining the intensified risk of AD among COVID-19 patients is associated with Aβ which has potential anti-microbial functions. As an immune response to the SARS-CoV-2 invasion into CNS, Aβ production and its cascade are augmented, resulting in Aβ deposition (Soscia et al., 2010). Moreover, when pericytes are lost and endothelial function is impaired, the clearance of cerebral metabolites, including Aβ peptides, is decreased leading to an excess of Aβ protein accumulation in senile plaques, particularly in the hippocampus (Ciaccio et al., 2021), which is the primary pathophysiological mechanism that causes AD. Additionally, hijack of the protein machinery by the virus and thus impairment of ER and mitochondrial functions can be another possible negative mechanism. This can propagate the aggregation of the misfolded proteins, which in turn, set out apoptosis and neurodegeneration (Scheper and Hoozemans, 2015; Dhungana and Jankovic, 2013; Wang et al., 2019).

One more variable which can be considered is that in critically severe COVID-19 patients, acute respiratory distress syndrome (ARDS) is accompanied by a prevalent long-term cognitive decline.

The standard therapy for this condition is mechanical ventilation, which leads to long-term cognitive impairment as well. It has been suggested that short-term mechanical ventilation may induce Aβ peptide accumulation in the brain, BBB impairment, neurologic and systemic inflammation, although the exact mechanisms are unknown (Zlokovic, 2004; Sasannejad et al., 2019; Montagne et al., 2017; van den Boogaard et al., 2011; Sharshar et al., 2004).

It is possible that pathogens can enter the CNS through peripheral nerve endings and lead to neurodegeneration, while gut microbiota dysbiosis may play a significant role in the spread of the virus and its invasion into the CNS (Dolatshahi et al., 2021). Studies have shown that some changes in the gastrointestinal tract (GI), such as GI lesions and increased permeability, occur decades before the onset of neurodegenerative diseases and may contribute to their development (Ambrosini et al., 2019). The alterations caused by gut microbiota dysbiosis can increase GI permeability, alter neurotransmission and lead to the activation of the immune system through mechanisms such as molecular mimicry and oxidative stress. These processes can then contribute to neurodegenerative disorders. Therefore, there is a possibility that SARS-CoV-2 infection, by modifying gut microbiota increases the risk of developing neurodegenerative diseases (Ambrosini et al., 2019). The diversity of gut microbiota is crucial for maintaining immunological balance and may impact susceptibility to infectious and inflammatory diseases such as COVID-19 (van der Lelie and Taghavi, 2020; Dhar and Mohanty, 2020). Older people, who are at greater risk of severe COVID-19 infection and neurodegenerative diseases, typically have less diverse gut microbiota (Dhar and Mohanty, 2020). Therefore, individuals who survive COVID-19 may have a higher risk of developing neurodegenerative conditions due to the common risk factor of reduced gut microbiota diversity (Dolatshahi et al., 2021).

The other influential mechanisms linking dysbiosis to neurodegenerative processes can be addressed by promoted intestine and BBB permeability, molecular mimicry, and oxidative stress which hyper-activate the immune system. These mechanisms disturb the neurotransmission balance and can cause neurodegenerative processes such as AD (Ambrosini et al., 2019). Intestinal bacteria produce short chain fatty acids (SCFAs) such as butyrate, folate, and thiamine, which are vital for maintaining the function of the epithelial barrier (Tan et al., 2014; Scheperjans et al., 2015). Long-term exposure to these SCFAs has been linked to clinical improvement in patients with PD, possibly due to ketogenesis (Luong and Nguyễn, 2013; Liu et al., 2017). Additionally, the similarity in structure between bacterial amyloid proteins and human Aβ can result in an increased inflammatory response to cerebral Aβ as a consequence of changes in the gut microbiota (Delzenne et al., 2011; Muegge et al., 2011; Rosenfeld, 2015). When lipopolysaccharide (LPS) aggregates, it can form structures that interact with various cellular components, leading to oxidative stress. This oxidative stress can, in turn, promote further protein aggregation, including amyloid species. The interaction between LPS and amyloid proteins can exacerbate the formation of insoluble aggregates, which are implicated in various neurodegenerative diseases (Schromm and Brandenburg, 2021).

Besides, one of the other determining factors which are common among severe COVID-19 patients can be disseminated intravascular coagulation (DIC) and hypercoagulability. These conditions can reduce perfusion, leading to ischemic white matter lesions. Admittedly, ischemic white matter damage is an early phase in AD patients and plays a role in AD progression and cognitive impairment. Cerebral hypoperfusion, in turn, can augment tau phosphorylation rate as well.

*APOE4* polymorphism is a proven predisposing factor for AD. It is worthy to mention that the growth of COVID-19 infection prevalence among people with homozygous *APOE e4* alleles has been reported. This genotype is mainly associated with an increased risk of severe COVID-19, independent of other comorbidities, as well. Thus, *APOE4* can be considered as a common risk factor for AD development and SARS-CoV-2 infection. Consequently, it is assumed that among genetically susceptible individuals, COVID-19 infection can be regarded as a factor accelerating neurodegeneration (Ciaccio et al., 2021; Kloske and Wilcock, 2020).

The last but not least probable mechanism linking AD to COVID-19 is that when SARS-CoV-2 binds to ACE-2, it can cause ACE-2 downregulation. Since ACE-2/ angiotensin (Agostini et al., 2019; Agostini et al., 2017; Bourgade et al., 2016; dos Santos Picanco et al., 2018; Breijyeh and Karaman, 2020; Kumar et al., 2015; Mawanda and Wallace, 2013)/Mas axis has neuroprotective functions, ACE-2 depletion can increase the risk of NDs development including AD (Ni et al., 2020). Therefore, according to the mentioned evidences, SARS-CoV-2 infection may worsen the AD development.

miRNAs can influence the expression of genes involved in both SARS-CoV-2 infection and AD. Research has identified common transcriptional signatures and pathways between COVID-19 and AD. miRNAs can target these pathways, potentially affecting the progression of both diseases. For example, miRNAs might modulate the PI3K-AKT, Neurotrophin, and JAK–STAT signaling pathways, which are implicated in both conditions (Premkumar and Sajitha, 2023). miRNAs can regulate neuroinflammatory responses, which are a key feature of both COVID-19 and AD. Moreover, exposure to pollutants, toxic metals, and endocrine-disrupting chemicals can alter the epigenetic regulation of key immune pathways (Bulka et al., 2022). This can increase susceptibility to SARS-CoV-2 and potentially exacerbate neurodegenerative processes associated with AD. Environmental factors can lead to changes in DNA methylation, histone modification, and miRNA expression (AbdelHamid et al., 2021). These epigenetic modifications can influence gene expression patterns, affecting both the immune response to SARS-CoV-2 and the progression of AD.

## Bacteria

### Chlamydia pneumonia (CP)

*Chlamydia pneumoniae* (CP) is an intracellular respiratory pathogen that can participate in the pathogenesis of pneumonia, multiple sclerosis (MS) and AD (Woods et al., 2020). Intravascular and olfactory pathways are the two means by which CP infects CNS. CP contaminates monocytes and human brain microvascular endothelial cells (HBMECs) in order to cross the BBB (Subedi et al., 2024). The infection of the HBMECs induces overexpression of surface adhesion molecules, intracellular adhesion molecule-1 (ICAM-1), vascular cellular adhesion molecule-1 (VCAM-1), VE-cadherin, N-cadherin, and β-catenin, and downregulates Occludin which is a tight junction membrane protein. These alterations increase the BBB permeability. Also, infection of the THP-1 monocytes upregulates integrin LFA-1 and MAC-1, the ligand of ICAM-1, and VLA-4, the ligand of VCAM-1, which promotes the transmigration of monocytes across the BBB (Itzhaki et al., 2004).

Transmigration of infected monocytes into the brain leads to the activation of astrocytes and glial cells, releasing pro-inflammatory cytokines including IL-1β, IL-6, and TNF-*α*, which trigger β-secretase and *γ*-secretase. Followingly, APP cleavage produces Aβ (Woods et al., 2020; Shima et al., 2010).

The exact mechanism by which CP infects the CNS is still unknown. Research suggests that *C. pneumoniae* can infect lung macrophages that move through the mucosal barrier and enter the bloodstream. The bacteria can then enter the vasculature by surviving intracellularly in blood monocytes, which can cross the BBB and spread to the CNS (Gieffers et al., 2004). However, other studies have proposed alternative routes of infection, such as the olfactory and trigeminal nerves that connect the nasal cavity to the brain. These structures are known to be a gateway for CNS infection by various pathogens. Interestingly, the structures of the CNS that show the earliest signs of pathology in AD (both familial and late-onset) are the olfactory bulb, entorhinal cortex, hippocampal formation, and brainstem, all of which are olfactory structures (Chacko et al., 2022).

Finally, there are five chlamydial phages identified from Microvicridae family viruses. They can enter the cells accompanied by CP. These phage’s DNA enters the mitochondria through transporter proteins and natural competence mechanism (Schmitz-Esser et al., 2004; Koulintchenko et al., 2006). Having entered the mitochondria, the DNA starts to transcribe as miRNAs. The phage’s miRNAs can interfere with human mRNAs and block their pathways (Dezfulian et al., 2008). They also infect the mitochondria and cause mutation in mitochondrial DNA and defects in mitochondrial oxidative phosphorylation enzymes (OXPHOS), including cytochrome C oxidase (Torrens-Mas et al., 2020). These mechanisms can result in the prohibition of ATP production, aggregation of dysfunctional proteins, production of chaperones, upregulation of the immune cells, and also the activation of the mitochondrial permeability transition pore (mtPTP) by escalating the production of ROS which contribute to apoptosis in the brain cells and cause AD (Dezfulian et al., 2008).

The pathogenesis of *Chlamydia psittaci* via miRNAs in human bronchial epithelial cells (HBE cells) has been investigated. Chen et al. found that *C. psittaci* induces oxidative stress in HBE cells and regulates the expression of miR-184 and FOXO1. MiR-184 was found to be significantly upregulated, and FOXO1 was confirmed as one of the target genes of miR-184 (Chen et al., 2023). The study also found that miR-184 can promote *C. psittaci*-induced oxidative stress in HBE cells by promoting the activity of the Wnt/β-catenin signaling pathway. Inhibition of the Wnt/β-catenin signaling pathway was found to reduce oxidative stress in *C. psittaci*-infected HBE cells. These findings highlight the importance of miRNA synthesis from viral structures in understanding the pathogenesis of *C. psittaci* (Chen et al., 2023).

### *Helicobacter pylori* (HP)

HP is classified as a gram-negative bacterium (Kountouras et al., 2007). It has an oral-fecal transition (Cuomo et al., 2020), colonizing the gastric mucosa and causing digestive disease and increasing the risk for vascular disorders and neurodegenerative diseases, such as AD (Kountouras et al., 2007). Douros et al. examined whether clinically apparent HP infection (CAHPI) is linked to AD. The population-based cohort included all UK Clinical Practice Research Datalink dementia-free subjects aged over 50 years, and matched each AD case with 40 controls. Results indicated that CAHPI was moderately associated with AD (11% increased risk) (Douros et al., 2024). Moreover, the nuclear magnetic resonance (NMR) of HP-infected cells has revealed excessive levels of branch chain amino acids (BCAA) isoleucine, leucine, and valine (Lynch and Adams, 2014). BCAA and glutamate metabolism have been found altered in AD patients (Lynch and Adams, 2014). The researches implicate that excessive levels of isoleucine and valine could play a role in the alleviation of AD risk (Tynkkynen et al., 2018). BCAA transmits across the BBB through the large neutral amino acid transporter (LAT 1) (Singh and Ecker, 2018). Although the chronically elevated BCAA plasma levels are the prime driving force for rising the BCAA uptakes of the brain, tryptophan, as an inhibitor for the production of serotonin, is prohibitive in the brain. Serotonin is a substance that alleviates the formation of Aβ and promotes neural survival. On the other hand, the branched-chain amino acid transaminase (BCAT) enzyme turns BCAA into glutamate. High levels of glutamate can induce neuronal death due to excitotoxicity. Furthermore, HP induces over-activation of mammalian target of rapamycin complex 1 (mTORC1) (Lynch and Adams, 2014). This activation could cause the breakdown of the BBB through endothelial cell dysfunction, as well as lead to tau hyperphosphorylation and the formation and aggregation of amyloid plaques in the brain. This is due to the inhibition of autophagy, which helps prevent the buildup of Aβ. When autophagy is inhibited, the aggregation of Aβ occurs (Mueed et al., 2018).

Moreover, HP induces AD by BBB disruption. The immune system produces TNF-*α* against HP and this is followed by over-activation of matrix metalloproteinase, leading to BBB disruption. However, HP itself produces vacuolating cytotoxin (VacA) which exerts bone marrow-derived mast cells (BMDMCs) and provokes them to produce pro-inflammatory cytokines such as vascular endothelial growth factor (VEGF), IL-8, chymase, and tryptase, therefore disturbing the BBB (Kountouras et al., 2012; Kountouras et al., 2007; Kountouras, 2009). HP infection has a likely effect on α-synuclein accumulation which can be transferred to the brain via blood or vagus nerve and exert microglial cells to release IL-1β. This process damages the BBB and provokes oxidative stress, causing degeneration of the neurons (Fu et al., 2020). Disruption of the BBB induces entrance of immune cells, including CD4+ and CD8^+^ T cells, and promotes their infiltration which results in brain tissue degeneration (Kountouras et al., 2012). Furthermore, HP releases acids such as (iso)valeric, (iso)butyric, propionic, acetic, and formic acid that can disturb the function of microglia and astrocytes, contributing to aggregation of Aβ and tau phosphorylation and thus the progression of AD (Kountouras et al., 2007). HP-n, a histidine-rich protein found abundant in HP, which plays a crucial role in the formation of amyloid-like oligomers, can cross the BBB through the main LRP-1 and RAGE. These are both transporters for Aβ in BBB, and have a potential role in causing AD through Aβ plaques mechanism (Kountouras et al., 2012; Zavos et al., 2012). Finally, an effective way to delay the AD progression could be the eradication of HP in the initial stages of AD (Kountouras et al., 2010). To clarify this point, Kountouras et al. have investigated 46 patients with AD, all tested by upper GI colonoscopy and bereaved from taking H2 receptor antagonists and proton pump inhibitors. During the 5-year investigation period, all participants received the same ChEI. At the end, AD patients with successful HP eradication survived 10.62 months longer than those patients whose eradication was unsuccessful (Kountouras et al., 2010).

### *Porphyromonas gingivalis* (*P. gingivalis*)

Periodontitis is a chronic inflammation leading to tooth loss and arousing the systemic inflammatory response. Various Gram-negative bacteria contribute to this inflammation, including *P. gingivalis*, and different viruses. *P. gingivalis* contains variety of virulence factors such as gingipains, outer membrane vesicles (OMVs) and cathepsin B (cat B) (Zhang et al., 2020). *P. gingivalis* has been detected in the brains of individuals with AD, alongside its harmful proteases, gingipains. This discovery correlates these levels with the existence of tau and ubiquitin pathology. Oral infection of mice with *P. gingivalis* has led to heightened production of Aβ1-42 and studies indicate that gingipains are detrimental to tau and possess neurotoxicity *in vivo* and vitro (Dominy et al., 2019). *In vivo* research has demonstrated that small molecule inhibitors of *P. gingivalis* gingipains can prevent neurodegeneration induced by gingipains and reduce *P. gingivalis* presence in the brain, whilst decreasing host Aβ1-42 response to *P. gingivalis* brain infection (Dominy et al., 2019).

*P. gingivalis* infects the oral cavity that is involved in the production of various pro-inflammatory molecules, including IL-1β, IL-6, and TNF-*α*. Besides, *P. gingivalis* accesses the brain through different mechanisms such as increasing the permeability of the BBB, pervading via cranial nerves like olfactory or trigeminal nerves and infecting the monocytes which are engaged by the brain (Dominy et al., 2019; Sadrameli et al., 2020). When *P. gingivalis* reaches the brain, gingipains, including arg-gingipain (Rgp) and lys-gingipain (Kgp), start the cleavage of pro-caspase-3 to produce activated caspase-3, a caspase which was associated with enhanced tau phosphorylation. This process impairs the neuronal function (Dominy et al., 2019; Costa et al., 2021). Furthermore, recent researches have shown that *P. gingivalis* OMVs carry out NLRP3 inflammasome activation and ASC speck accumulation and cause Aβ formation, inducing pre-apoptosis and neural cell death (Dominy et al., 2019).

LPS is a crucial part of the structure of OMVs in Gram-negative bacteria. When *P. gingivalis* LPS/lipoprotein interacts with pattern recognition receptors on innate immune cells, it can trigger immune responses. This can also lead to neuroinflammation in microglia-dependent manner. There is some debate, but evidence suggests that TLR2 and/or TLR4 on microglia may be the targets for *P. gingivalis* LPS/lipoprotein preparations. From here, downstream NF-κB and STAT3 pathways are activated, leading to increased expression and secretion of pro-inflammatory cytokines, including TNF-a, IL-1β, IL-6, IL-17 and IL-23 (Nativel et al., 2017; Qiu et al., 2018). *P. gingivalis* LPS preparations induce cathepsin B in microglial cells through the NF-κB pathway, leading to IL-1β production by microglia. When released, IL-1β acts on neuronal IL-1 receptors which promotes *APP* expression and tau phosphorylation (Villemagne et al., 2013). This further activates the NF-κB pathway and promotes microglia-mediated neuroinflammation (Wu et al., 2017). Moreover, *P. gingivalis* LPS preparations stimulate GSK-3b activation in microglia leading to the expression and secretion of TNF-a that acts on neurons and promotes AKT-GSK-3b-mediated tau hyperphosphorylation (Jiang et al., 2021). Conversely, *P. gingivalis* LPS treatment has been shown to upregulate the inactive form of protein phosphatase 2, a principal phosphatase for tau de-phosphorylation, rather than directly change GSK-3b activity in an APP-over-expressing neuroblastoma cell line (Zeng et al., 2021). A recent transcriptome study found that *P. gingivalis* LPS treatment of human neuroblastoma cells affects an array of interconnected pathways involving cellular oxidative stress, inflammation, and metabolism (Bahar and Singhrao, 2021). All these findings suggest a complex role for *P. gingivalis* LPS in AD pathophysiology, much like its role in the pathology of periodontitis.

As mentioned before, chronic periodontitis causes tooth loss in adults. Having fewer teeth leads to a reduction in chewing and also decreases acetylcholine level in the hippocampus through degeneration of pyramidal cells, resulting in memory loss (Gaur and Agnihotri, 2015). It should be noted that periodontitis caused by *P. gingivalis* can leads to the overexpression of platelet aggregation proteins and atherosclerosis. Moreover, a systemic inflammation caused by this pathogen may be contributed in endothelial dysfunction. So, considering the accumulating evidence suggesting the role of vascular dysfunction in the pathogenesis of AD, the periodontitis caused by *P. gingivalis* may have a contribution role in the development of AD (Uppoor et al., 2013). *P. gingivalis* infection induces changes in the molecular clock function of the microglial cells. Followingly, this impairs the sleep patterns and circadian system which is known as an important factor in the phagocytosis activity of microglial cells. Therefore, this process can lead to decreased clearance of Aβ peptides and an elevation in accumulation of aggregated proteins (Harding et al., 2017). The glymphatic system, which uses glial water channels to clear interstitial solutes and Aβ plaques from the brain (Xie et al., 2013), is “turned on” during normal sleep and reduced during the awake state (Xie et al., 2013). With aging and mixed pathological factors, intrinsic stress due to infections, which affects sleep quality and duration, impairments to the glymphatic system may interact to influence AD development and progression (Slats et al., 2013; Ju et al., 2013). *P. gingivalis* can disturb the microglial cell phagocytic activity by disrupting the circadian system that controls sleep–wake cycles (Takayama et al., 2016). Consequently, the glymphatic system appears less efficient, and this may lead to aggregated protein build-up. This connection has crucial relevance to sleep pattern disturbances in AD and supports how poor oral hygiene and rising levels of intrinsic and extrinsic sources of cytokines may act as crucial early modifiers of neurodegeneration and disease severity leading to deteriorating memory, sleep, and ultimately the development of pathology (Harding et al., 2017).

The pathogenesis of *P. gingivalis* via miRNAs in human periodontal ligament cells (hPDLCs) has been explored. Fan et al. found that *P. gingivalis* OMVs promote alveolar bone resorption *in vivo* and decrease cell viability in hPDLCs by inducing apoptosis and inflammation (Fan et al., 2023). Transcriptome sequencing results showed that *P. gingivalis* OMVs were involved in gene regulation, mRNA processing, endocytosis, ubiquitination, and the cell cycle process, and several small RNAs secreted via *P. gingivalis* OMVs were identified, including sRNA45033. This small RNA directly bound to the 3′ UTR of the downstream target gene *CBX5*, resulting in decreased levels of *CBX5* in *P. gingivalis* OMV-stimulated hPDLCs, which regulated apoptosis through p53 and H3K9me3 methylation (Fan et al., 2023).

### Spirochetes

Spirochetes, Gram-negative, helical bacteria have been found in various human tissues such as mouth, genital mucosa, and GI tract. The spirochete *Borrelia burgdorferi* is a tick-borne agent that causes Lyme disease. The symptoms include influenza-like illness and neurological indications. Many studies have indicated that there is an association between spirochetes and their induced illnesses, especially Lyme disease with AD (Miklossy, 2008). This relationship develops through various mechanisms. Studies demonstrate that the outer surface protein (Osp A) of spirochetes is an amyloid executor that leads to Aβ formation and thus AD (Miklossy, 2008). Furthermore, B.burgdorferi evades the immune system through binding to the complement inhibitor factor H (FH) and factor H like protein-1 (FHL_1) (Dulipati et al., 2020). Through this mechanism the bacterium is protected against phagocytosis and complement lysis and thus continues to survive and reach the brain via blood circulation. It can cross the BBB and proliferate in the infected tissue (Miklossy, 2008). When they reach a quorum, they generate a biofilm. Production of the biofilm protects the microorganism against the immune system (Rutherford and Bassler, 2012). The first factor which responds to this organism is TLR2. TLR2 induces the release of TNF-α and NF-κB through myeloid differentiation pathway D88 (MYD88) (Allen et al., 2016). TNF-α, in combination with TNF-α converting enzyme (TACE), forms the α-secretase to produce amyloid alpha. Thus, NF-κB and Aβ converting enzyme (BACE) activate β- and *γ*-secretase. This process finally leads to the production of Aβ which attacks the biofilms but is not able to annihilate them, instead cause the destruction of the surrounding tissue (Allen, 2016). Besides, iron is a necessary growth factor for the bacterium. The aggregation of the iron in the brain accelerates the production of reactive oxygen which leads to lipid peroxidation and induces oxidative stress (Carocci et al., 2018). Iron also induces T cells to produce pro-inflammatory cytokines (Griffiths, 1991; Weinberg, 1992). Both of these processes lead to Aβ deposition and the pathogenesis of AD (Miklossy, 2008).

Generally, B.burgdorferi may cause AD through its various surface antigens such as Osp A, producing a biofilm to protect them against the immune system and aggregating iron for its division.

## Parasite

### *Toxoplasma gondii* (*T. gondii*)

*T. gondii* is a protozoan parasite belonging to the phylum Apicomplexa. Research has indicated that the parasite *Toxoplasma gondii* (*T. gondii*) impairs learning and memory functions. However, interestingly, the same studies have also reported that this infection enhances synaptic plasticity in the dorsal hippocampus. This dual effect suggests a complex interaction between the parasite and the host’s neural mechanisms. On one hand, the cognitive deficits highlight the detrimental impact of *T. gondii* on overall brain function. On the other, the increase in synaptic plasticity points to a paradoxical enhancement in the adaptability and connectivity of neurons within the dorsal hippocampus, which is critical for memory formation and spatial navigation (Choopani et al., 2023). Possible involvement of *T. gondii* in the progression of AD could be due to various mechanisms, including its ability to induce the host immune responses, cause inflammation in the central nervous system, alter neurotransmitter levels, and activate indoleamine-2,3-dyoxigenase (Nayeri et al., 2021). The development of AD is influenced by neuroinflammation, oxidative stress, and vascular factors (Aliev et al., 2014). Inflammation has been identified as a factor in AD for almost 20 years (Kusbeci et al., 2011). Taking nonsteroidal anti-inflammatory drugs can reduce the risk of developing AD (Vlad et al., 2008). Inflammatory responses are also the innate defense against *T. gondii* infection (Guerreiro et al., 2013; Lambert et al., 2010). *T. gondii* triggers the immune system to release cytokines such as IFN-γ, IL-12, IL-1, IL-6, and TNF (Kusbeci et al., 2011; Glass et al., 2010). While these cytokines can protect against *T. gondii*, they can also damage non-infected neurons and affect neurotransmitter function and synaptic transmission (Dunn, 2006; McCusker and Kelley, 2013). Inflammatory mediators can stimulate the progression of AD by activating the processing of APPs (Griffin and Barger, 2010). During toxoplasmic encephalitis, the activity of neurotransmitters is affected by cytokines and inflammatory mediators through various mechanisms including activation of indoleamine-2,3-dioxygenase enzyme, activation of mitogen-activated protein kinase pathways, changes in tetrahydrobiopterin enzyme activity, excitotoxicity, and oxidative stress (Haroon et al., 2012). Moreover, patients with AD exhibit high levels of the transcription factor NF-κB (Camandola and Mattson, 2007), which modulates immune and inflammatory responses and is activated during *T. gondii* infection (Gupta et al., 2010; Blader and Saeij, 2009). The activation of NF-κB can accelerate neuroinflammation to neurodegeneration in AD (Srinivasan and Lahiri, 2015), but it also prevents the apoptosis of infected cells (Molestina and Sinai, 2005). Inhibition of NF-κB target genes involved in inflammation can disrupt the immune response to *T. gondii*, allowing the parasite to replicate (Molestina and Sinai, 2005).

In addition, infection with *T. gondii* increases the INF-*δ* secretion and leads to NO production which causes neural degeneration resulting in AD (Nayeri Chegeni et al., 2019; Mahami-Oskouei et al., 2016). Luisa Torres et al. infected wild-type mice with *T. gondii* and tested their anatomical and behavioral impressions via immunohistochemistry, western blotting and immunofluorescence. They claimed that *T. gondii* disturbs the N-methyl-D-aspartate receptor (NMDAR) signaling which plays a vital role in synaptic plasticity. This destruction impacts Aβ accumulation and hyperphosphorylation of tau which consequently leads to AD (Torres et al., 2018).

On the other hand, some studies stated that Toxoplasmosis cannot be considered as a risk factor for progression of AD (Nayeri Chegeni et al., 2019). A meta-analysis study conducted by Mahami-Oskouei et al. (2016) reported that *T. gondii* cannot be considered as a risk factor for AD progression (Mahami-Oskouei et al., 2016). Accordingly, a number of researches have suggested that AD could have an adverse effect on progression as a result of *T. gondii* infection. Toxoplasmosis promotes expression of anti-inflammatory responses such as suppressor of cytokine signaling 1(SOCS1), Arg1, TGF-β and IL-10, and also decreases the generation of inflammatory mediators including NO (Jung et al., 2012; Rozenfeld et al., 2005; Cabral et al., 2017). Furthermore, Jung et al. (2012) indicated that *T. gondii* infection causes immunosuppression in its hots and inhibits AD progression. They infected mice with *T. gondii* and assayed anti-inflammatory cytokines, including IL-10 and TGF-β, and also Aβ accumulation in mice’s brain and used water maze and Y-maze behavioral tests. The results showed that anti-inflammatory cytokines levels were remarkably higher, while Aβ plaques deposition was significantly lower, in both the hippocampus and cortex of *T. gondii* infected mices (Jung et al., 2012). Furthermore, *T. gondii* prohibits apoptosis by upregulation of anti-apoptotic genes or by disturbing the apoptotic signaling pathway. Exposure to *T. gondii* promotes M1 polarization of microglia and microglial proliferation. The accelerated proliferation of microglial cells leads to Aβ phagocytosis and clearance. These processes prevent neural degeneration and AD (Shin et al., 2021).

Generally, infection with *T. gondii* may lead to AD through increasing inflammatory mediators, NO production, disrupting synapses and Aβ plaque deposition. On the other hand, due to the augment of anti-inflammatory cytokines, it presumably inhibits the neuronal degeneration. At last, more studies are needed in this area.

## Conclusion

Given the literature, infectious agents are likely to play a profound role in the pathogenesis of some neurodegenerative diseases like AD. In this review, the effect of various pathogens in AD pathogenesis have been reviewed. Several studies have suggested that infectious agents can foster the Aβ cascade. Additionally, brain infection can trigger tau protein hyperphosphorylation, which results in neuronal degeneration and loss of synapses. Furthermore, both local and systemic infections can induce microglial and astrocytic activation and the release of pro-inflammatory cytokines by over-stimulating the immune system. This process has been consistently associated with excessive oxidative stress, which has emerged as a significant factor in AD pathogenesis. Genetic predispositions, immune response, lifestyle factors, and microbiome balance can all play a role in the development of AD. Aging and other health conditions can also increase vulnerability to the disease. However, not every infected person develops AD, as individual factors may influence outcomes. A robust immune system and healthy lifestyle choices can help mitigate the impact of infections on brain health. Understanding the specific mechanisms by which these pathogens influence Aβ deposition and neuroinflammation can open new avenues for therapeutic interventions. Further research is essential to elucidate the precise roles of these pathogens in AD and to develop effective treatments that address the multifaceted nature of this devastating disease.
